# Mental Health Disorders Due to Disaster Exposure: A Systematic Review and Meta-Analysis

**DOI:** 10.7759/cureus.37031

**Published:** 2023-04-02

**Authors:** Tahmina A Keya, Anthony Leela, Nasrin Habib, Mamunur Rashid, Pugazhandhi Bakthavatchalam

**Affiliations:** 1 Community Medicine, Mahatma Gandhi Medical College and Research Institute, Pondicherry, IND; 2 Community Medicine, Asian Institute of Medicine, Science and Technology (AIMST) University, Bedong, MYS; 3 Physiology, Quest International University, Perak, MYS; 4 Medicine, Quest International University, Perak, MYS; 5 Anatomy, Quest International University, Perak, MYS

**Keywords:** catastrophic, disruption, relocation, post-traumatic stress disorder, mental health illness, natural disaster, depression, anxiety

## Abstract

Natural disasters are complex, global issues that affect people individually, families, and communities, upsetting their emotional wellbeing. This research aims to comprehend the connections between disasters and their effects on mental health. We conducted a systemic review and meta-analysis on the effect of disasters on mental health disorders using defined search terms across three major databases. The search technique adhered to the PECO framework. The study locations were dispersed across Asia, Europe, and America. An electronic search was established in the Cochrane Central Register of Controlled Trials in the Cochrane Library, PubMed, and Medline databases. A random-effects meta-analysis was carried out. The I^2^ statistic was used to explore heterogeneity. In the random-effects analysis, Tau-squared, τ^2^, or Tau^2^ evaluates the effects seen between the study variances. Publication bias was examined. The outcomes of the included studies on mental health issues (n = 48,170) brought on by catastrophic disasters were pooled using a random-effects meta-analysis. The three main mental health illnesses attributed to the disaster catastrophe in most studies were generalized anxiety disorder (GAD), depression, substance use, adjustment disorder, and post-traumatic stress disorder (PTSD). Storms, including cyclones and snowstorms, had an impact on 5,151 individuals. 38,456 people were harmed by flooding, and 4,563 people were affected by the earthquake. The included studies showed prevalence rates for mental health disorders ranging from 5.8% to 87.6%. The prevalence rates were between 2.2% and 84% for anxiety, 3.23% and 52.70% for depression, and 2.6% and 52% for PTSD, respectively. The point effect estimates of studies included the flood, storm/cyclone, and earthquake were 0.07 (95% confidence interval [CI]: 0.02-0.12), 0.18 (95% CI: 0.03-0.32), and 0.15 (95% CI: 0.03-0.27), respectively, which revealed a statistically significant positive effect (p-value: < 0.05) with a narrow 95% CI indicating more precise population estimates. However, the pooled effect estimates were not of a large effect size of 0.129 (95% CI: 0.05-0.20). This study found a link between disaster and poorer outcomes for mental health. The risk of psychological morbidity and fatalities increased with relocation and disruption of essential services. Flooding was the most frequent calamity. The “medium human development countries” were found to have the highest prevalence rate of mental health disorders in our meta-analysis. The “very high human development” and “high human development” nations, however, also had a higher prevalence rate of mental health disorders following catastrophic events. This study could aid in the creation of thorough strategies for the mitigation and avoidance of mental health problems during natural disasters. Increased community resilience, improved access to healthcare services, and a suitable mitigation strategy can all help to improve the situation of the disaster's vulnerable population.

## Introduction and background

Key message

-Natural disasters and poorer mental health outcomes are linked.

-With the relocation and the disruption of vital services, the risk of psychological morbidity and fatalities rises.

-PTSD is correlated with ill health, high exposure, previous traumatic experiences, aging, and property damage.

-Lower socioeconomic status is linked to persistent psychological anguish.

-Regional differences may exist in how a catastrophe affects mental health.

Natural catastrophes are an unavoidable reality of life and a complex worldwide concern. Disasters afflict people and communities every year, which undermines their mental health and well-being [1]. Disaster is defined by the World Health Organization (WHO) as a sudden ecological catastrophe or a phenomenon that necessitates outside help [2]. The UN Office for Disaster Risk Reduction (UNDRR) defines a disaster as a serious disruption of a community's or society's functioning at any scale caused by hazardous events interacting with conditions of exposure, vulnerability, and capacity, resulting in one or more of the following: human, material, economic, and environmental losses, and impacts [3]. Natural disasters are brought on by floods, cyclones, earthquakes, tsunamis, and tropical cyclones [4].

According to the Global Burden of Disease survey, the prevalence of mental health disorders accounts for more than 10% across all continents [5]. Disasters increase the likelihood of negative mental health effects like severe posttraumatic psychopathologies. The outcomes of catastrophic disasters are worse when they occur in poorer nations [6,7].

In this article, we assess the prevalence of mental health disorders in populations exposed to natural disaster occurrences across different continents. To do this, we conducted a systematic review and meta-analysis. In Western populations, post-disaster research has received a lot of attention. In developing nations, very little research has been conducted. The current status of research in this area still has a significant gap [8,9]. This study offers a thorough review of the literature on the effects of major catastrophes on mental health. This research may contribute to the development of comprehensive approaches for the prevention and mitigation of mental health issues during times of natural disaster.

## Review

Methods

This study strictly complies with the reporting guidelines for systematic reviews (PRISMA) [10], and our study protocol was previously applied for registration (30.12.2022; PROSPERO) [11].

Search Strategy and Selection Criteria

The search technique adhered to the PECO framework: Participants, Exposure, Comparator, and Outcomes. The study populations included the UNDP human development groups of very high human development, high human development, medium human development, and low human development categories [12]. The study locations were dispersed across Asia, Europe, and America. Natural disasters like floods, storms, cyclones, etc., served as the exposure, and the study outcome was mental health illnesses including PTSD, depression, and anxiety.

We adopted the WHO definition of “mental health disorder” for this review, which defined mental illnesses as psychological discomfort, obsessive-compulsive disorder, phobias, panic disorder, post-traumatic stress disorder (PTSD), and depression [13]. The definition of some key meteorological terms, including “natural catastrophes,” “flood,” “storm,” “snowstorm,” and “cyclone,” was taken from the UNDRR report [3].

An electronic search was established in the Cochrane Central Register of Controlled Trials (CENTRAL; Issue 11 of 12, November 2022) in the Cochrane Library, PubMed, and Medline databases (Searched December 27, 2022). We looked for the health outcome phrases “mental disorder” and “mental illness” along with the disaster terms “natural disaster,” “flood,” and “storm” in electronic databases. In addition, we looked for other pertinent publications in the 24 review articles' references that were found during the search.

The observational research, which included cross-sectional and cohort studies, was eligible for this analysis. Only published research was incorporated. Studies were considered if they fulfilled the following requirements: The study covered common mental health issues as listed in the ICD-10 tenth edition [14]; mental health outcomes were assessed using validated self-report scales or checklists, such as the Generalized Anxiety Disorder scale (GAD-2), (GAD-7), (K-6), the Patient Health Questionnaire (PHQ-2), (PHQ-9), depression sub-scale, and the short-form PTSD checklist (PCL-6), (IES-R). Experiments that were conference abstracts, reports, reviews, meta-analyses, letters, pilot studies, or procedures were excluded. Studies in the English language on the effect of disaster on mental health mortality or morbidity that were published between 1985 and 2022 in peer-reviewed publications were retrieved. Duplicates were removed, and titles and abstracts were evaluated for compliance with the inclusion and exclusion criteria, and the full text of the potentially relevant references was assessed. TK and NH implemented the search strategy. MR and PB provided clarification on any questions surrounding the inclusion of papers. Any disputes were settled by contacting two experienced investigators (AL and MR). AL checked the datasets after TK had assembled them. With advice and feedback from AL, TK carried out the statistical analysis. NH, MR, and PB contributed their statistical skills. AL crosschecked the findings of the statistical analysis.

**Figure 1:**
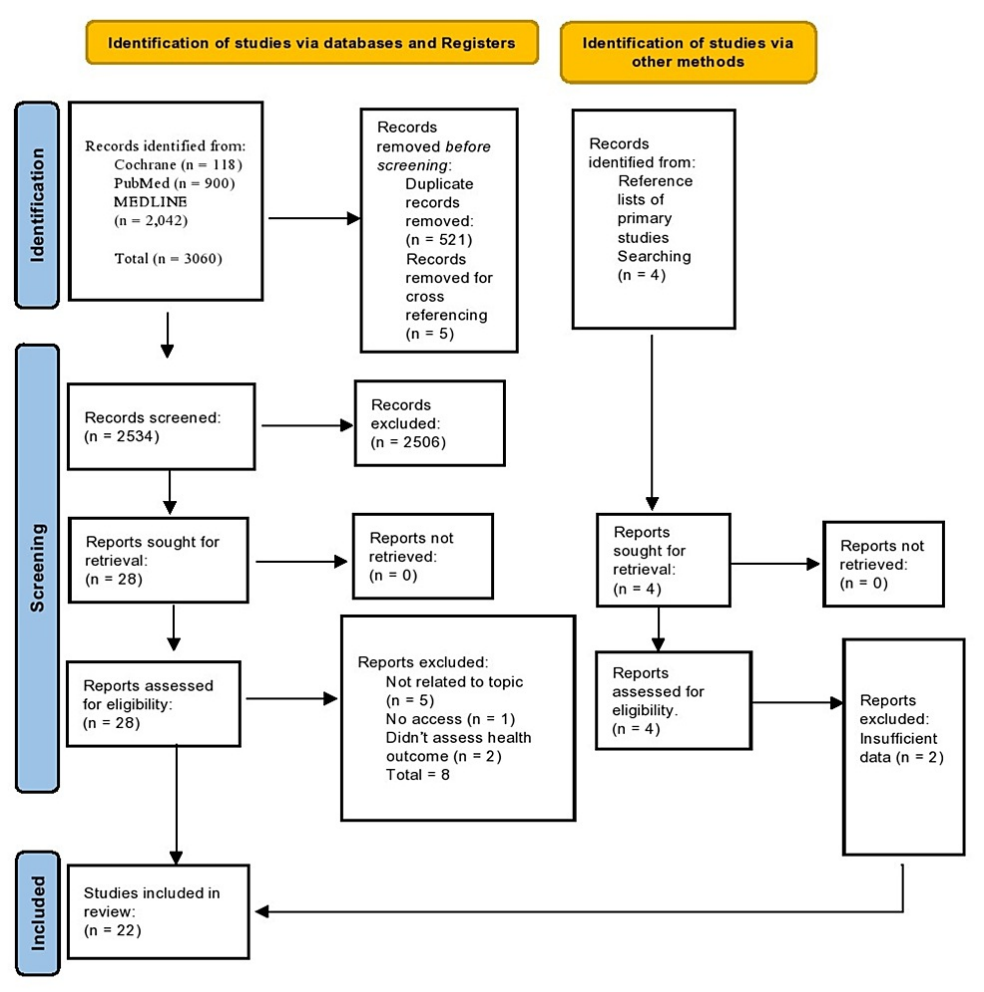
PRISMA flow diagram of study selection

The systematic search retrieved 3,060 studies that related to natural disasters impact on the mental health outcomes. Twenty-two publications from the 28 studies that met the inclusion and exclusion criteria were suitable for this meta-analysis (Figure 1).

A random-effects meta-analysis was carried out. The I^2^ statistic, which values can range from 0% to 100%, was used to explore heterogeneity [15]. In the random-effects analysis, Tau-squared, τ^2^, or Tau^2^ evaluates the effects seen between the study variances [16].

Publication bias was also examined. We assessed the studies’ risk of bias in accordance to the Cochrane Handbook for Systematic Reviews of Interventions [17]. The risk-of-bias domains were divided into three categories: low risk, uncertain risk, and high risk of bias. The Review Manager (v-5⸳4⸳1⸳0) [18], and jamovi (v-2.3.13) software were used for the data analysis.

Study Characteristics

The 22 included studies examined the impact of catastrophic natural disasters such as flood, storm, super-cyclone, typhoon, hurricane, snowstorm, and earthquake on a range of mental health outcomes such as anxiety, depression, post-traumatic disorder syndrome (PTSD), and psychological distress (Table 1).

**Table 1:** Summary of included studies PTSD: post-traumatic stress disorder, MDD: major depressive disorder, GAD: general anxiety disorder, PTSS: post-traumatic stress symptoms

Study	Study design	Location	Event	Year of Event	Time point Measured (month)	Outcomes Assessed	N
Munro et al 2017 [19]	Cross-Sectional	England	Flood	2013-14	12	Depression, Anxiety, PTSD	605
Jermacane et al 2018 [20]	Cohort	England	Flood	2013-14	24	Anxiety, Depression, PTSD	988
Graham et al 2019 [21]	Cross-Sectional	England	Flood	2013-14	6	Depression, Anxiety, Obsessive compulsive disorder, panic disorder; Phobias, PTSD, Suicide ideation.	7525
Mason et al 2010 [22]	Cross-Sectional	England	Flood		6	Depression, Anxiety; PTSD.	444
Kar et al 2004 [23]	Cross Sectional	India	Storm, super-cyclone	1999	5	PTSD, Anxiety, Depression	540
Paranjothy et al 2011 [24]		South Yorkshire and Worcestershire	Flood	2007	6	psychological distress, anxiety, depression, PTSD	2113
Tunstall et al 2006 [25]	Cross-Sectional	England and Wales	Flood	1998	60	Anxiety, Depression, PTS, Psychological distress, Suicide ideation	982
Reacher et al 2004 [26]	Cohort Study	Lewes	Flood	2000	9	Psychological distress	321
Caldera et al. 2001 [27]	Cross Sectional	Nicaragua	Storm, Hurricane Mitch	1998	6	PTSD	496
Huang et al. 2010 [28]	Cross Sectional	China	Flood	1998	24	PTSD	25478
Kar & Bastia 2006 [29]	Cross Sectional	India	Storm, super-cyclone	1999	14	PTSD, MDD, GAD	108
Kar et al. 2007 [30]	Cross Sectional	India	Storm, super-cyclone	1999	12	PTSD	447
Kohn et al. 2005 [31]	Cross Sectional	Honduras	Storm, Hurricane Mitch	1998	2	PTSD, Depression	800
Norris et al. 2006 [32]	Cross Sectional	Mexico	Flood due to storm	1999	6	PTSD	666
Wu et al. 2011 [33]	Cross Sectional	China	Storm, snowstorm	2008	3	PTSD	968
Amstadter et al. 2009 [34]	Cohort	Vietnam	Storm, Typhoon	2006	3	PTSD, MDD, GAD	797
Patrick & Patrick 1981 [35]	Cross Sectional	Sri Lanka	Storm, cyclone	1978	1	Anxiety, Depression	171
Goenjian et al. 2001 [36]	Cross Sectional	Nicaragua	Storm, Hurricane Mitch	1998	6	PTSD, Depression	158
Honda et al., 2019 [37]	Longitudinal studies	Japan	Earthquake	2011	24-36	PTSD	314
Kino et al., 2021 [38]	longitudinal cohort	Japan	Tsunami, Earthquake	2011	36	Depression PTSS	4010
Schwind et al., 2018 [39]	Cross‑ sectional	Nepal	Earthquake	2015	12	Depression, PTSD,	62
Valladares-Garrido et al., 2022 [40]	Cross-Sectional	Peru	Earthquake	2021	1-2	Depressive and Anxiety	177

Result

The study examining the impact of catastrophic natural disasters on mental disorders. Data were extracted and checked for each of the 22 included studies. 5,151 people were affected by storms, including cyclones, super-cyclones, typhoons, hurricanes, and snowstorms. Flooding, earthquake affected 38,456, and 4,563 individuals, respectively (Table 1).

**Figure 2:**
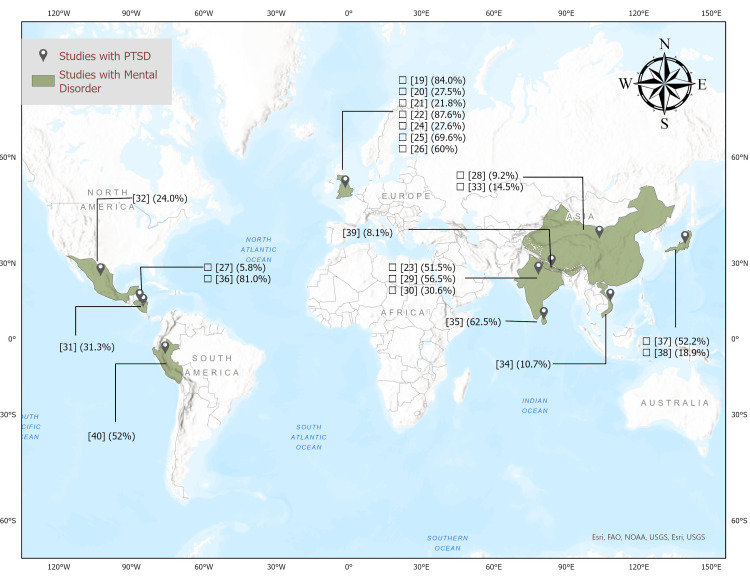
Distribution of studies with mental health disorders Study Reference numbers on the map are defined in Table 1. (This map was created by using Arcgis software.)

Overall, Caldera et al. [27] and Mason et al. [22] reported the lowest (5.8%) and highest (87.6%) prevalence rates of the mental health disorder, respectively. Anxiety and GAD were detected in all 22 studies, while 19 of them reported having had PTSD. The included studies showed prevalence rates for the mental health conditions ranging from 5.8% to 87.6%. The prevalence rates were between 2.2% and 84% for anxiety, 3.23% and 52.70% for depression and 2.6% and 52% for PTSD, respectively (Table 1, Figure 2).

The three main mental health illness attributed to the disaster catastrophe in most studies were anxiety, GAD, and PTSD [19-22,24,25,32,37-40]. In none of the references was it noted that the responders had mental health issues prior to the crisis occurring.

Participants in the study reported having physical ailments as well as problems with their mental health illness [25,26]. Existing psychological distress was increased by poor water quality and the potential for flood water pollution [24-26]. Flooding had a long-lasting effect that caused more people to seek medical attention for psychological distress between six and 24 months following the catastrophic occurrence [25,26,41] According to reports, flood victims have an increased risk of long-term mental health issues of four [26] to eight times [20] that of non-flooded people. Years after the incidence, those who had experienced floods still experienced anxiety during heavy rain [25]. Some of our included studies showed a connection between PTSD, depression, and anxiety symptoms after the earthquake [37-40].

Disasters affected people in different ways over time, some reported increased PTSD and anxiety symptoms [22]. However, others asserted that exposure to a single event or repeated ones increased the chance of psychological morbidity in a similar manner [42]. Most of the studies revealed that temporary accommodation and evacuation increased psychological suffering, including anxiety, depression, and PTSD. Due to the disruption of necessary services, job, or education, an increase in mental illness was observed in addition to relocation [24,37-40]. Respondents who reported persistent property damage were more likely to experience depression and anxiety than those who did not [20,37-40].

**Figure 3:**
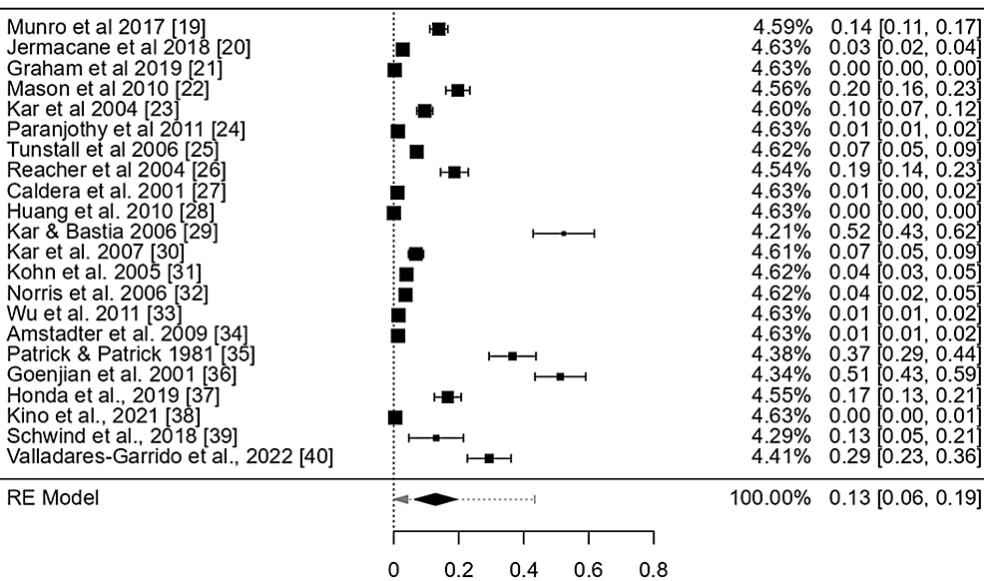
Point and pooled effect estimates of the mental health disorders after disaster exposure across 22 countries Estimated using random effects model.

The overall pooled effect estimates for mental health disorders related to natural disasters across 22 countries, was 0.13 (95% confidence interval (CI): 0.06-0.19) (Figure 3), which was not a large effect size.

**Figure 4:**
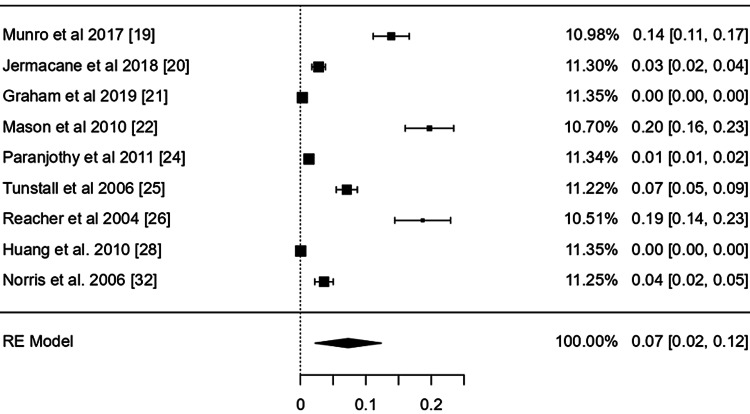
Point effect estimates of the mental health disorders after flood disaster exposure across nine countries Estimated using random effects model.

The point effect estimates from nine studies [19,21,22,24-26,28,32,41] of flood affected people, nine studies [23,27,29-31,33-36] of storm/cyclone , and four studies [37-40] of earthquake were 0.07 (95% CI: 0.02-0.12), 0.18 (95% CI: 0.03-0.32), and 0.15 (95% confidence interval (CI): 0.03-0.27) respectively which revealed a statistically significant positive effect (p value: < 0.05) with a narrow 95% CI indicating more precise population estimates (Figure 4).

**Figure 5:**
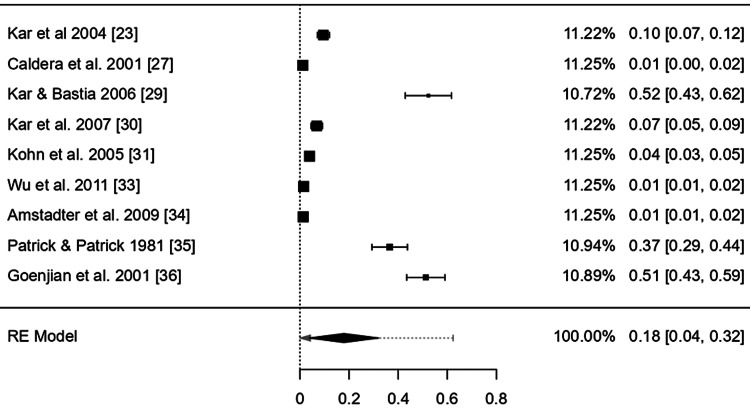
Point effect estimates of the mental health disorders after storm/cyclone exposure across nine countries (Estimated using random effects model.)

**Figure 6:**
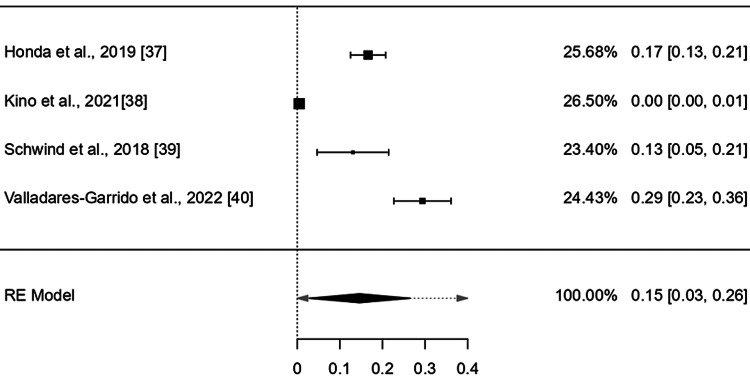
Point effect estimates of the mental health disorders after earthquake exposure across four countries (Estimated using random effects model.)

The CI of the combined effect size did not include null, demonstrating that the meta-analytic effect is statistically significant (p-value: < 0.001). The heterogeneity was, however, considerable: I^2^ = 99.99%%, 𝜏^2^ = 0.02 (Figures 3-6).

Publication Bias

**Figure 7:**
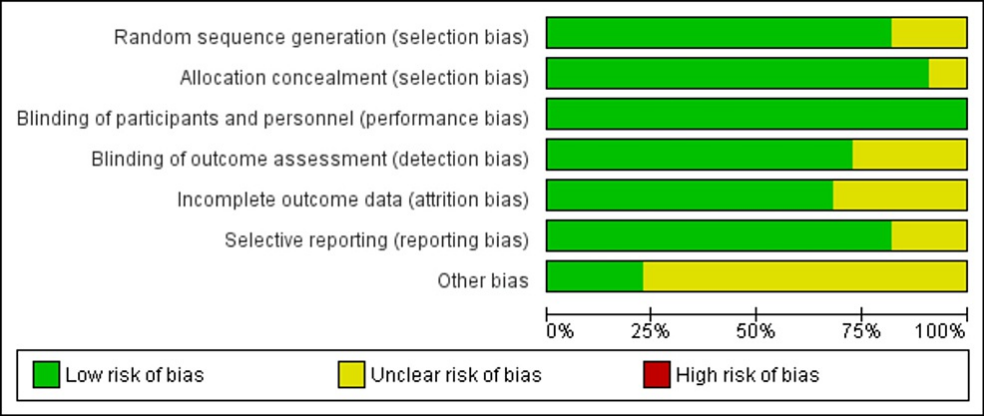
Risk of bias graph: percentages across all included studies.

**Figure 8:**
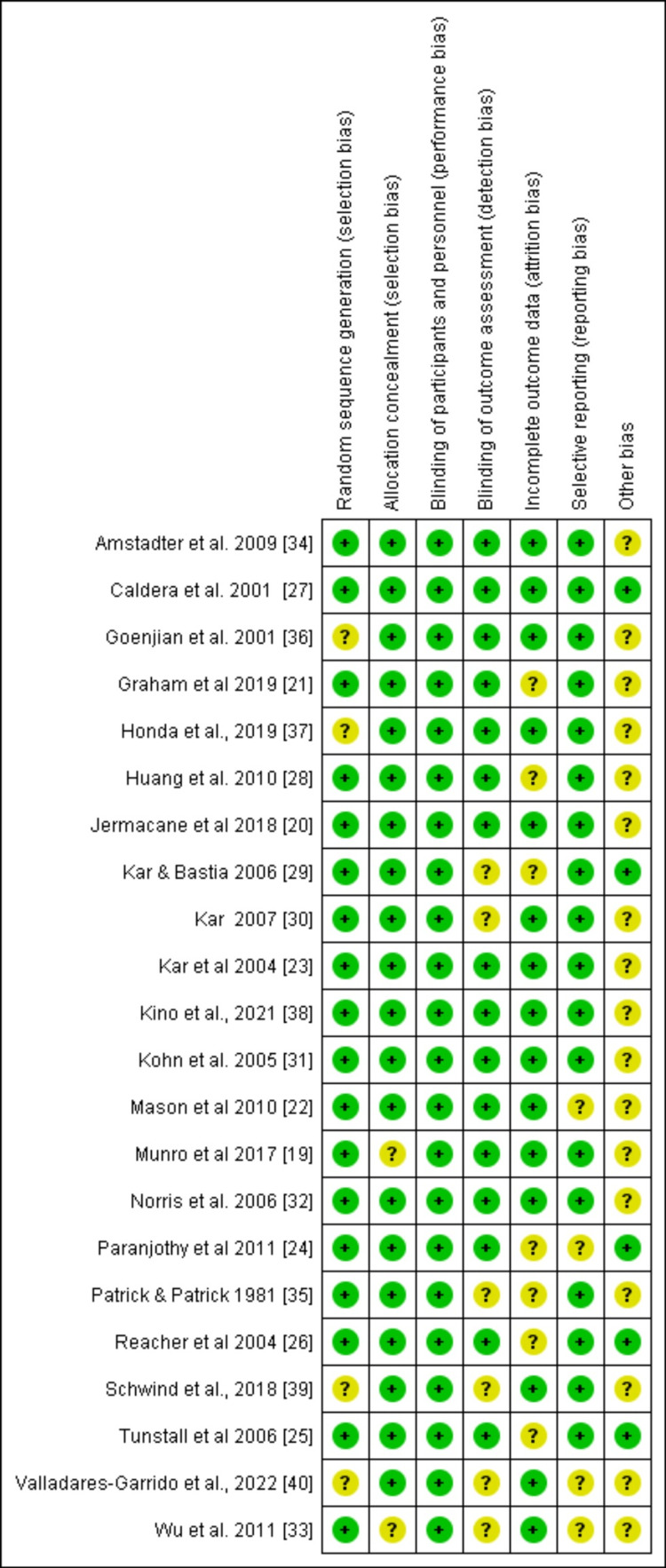
Risk of bias summary.

Overall, the risk of bias in the included studies was low. Most studies used randomized sample techniques (Figures 7, 8).

Allocation concealment was used in most of the studies. A recall, information, measurement, and confounding bias may have occurred because of the length of time following the disaster's impact, the potential for confounding the primary interest's exposure with unrelated risk factors, and the difference in participation rates between households that were affected and those that were not. The study's findings, nevertheless, should not be impacted. However, Fail-Safe N Analysis using the Rosenthal approach and rank correlation test for funnel plot asymmetry were significant (p < 0.05).

Discussion

Our systematic review and meta-analysis reveal catastrophic disaster occurrence is associated with an increase in risk mental disorders in the general population. The dataset comprises 48,170 participants.

The three main mental health illness attributed to the disaster catastrophe in most studies were GAD, depression, substance use, adjustment disorder, and PTSD. The included studies showed prevalence rates for the mental health disorder ranging from 5.8% to 87.6%.

The point effect estimates of studies included the flood, storm/cyclone, and earthquake were 0.07 (95% CI: 0.02-0.12), 0.18 (95% CI: 0.03-0.32), and 0.15 (95% CI: 0.03-0.27) respectively which revealed a statistically significant positive effect (p value: < .05) with a narrow 95% CI indicating more precise population estimates. However, the pooled effect estimates were not of a large effect size 0.129 (95% CI: 0.05-0.20).

This study found a link between disaster and poorer outcomes for mental health. The risk of psychological morbidity and fatalities increased with relocation and disruption of essential services. Flooding and storm/cyclones were the most frequent calamities. The “medium human development countries” were found to have the highest prevalence rate of mental health disorders in our meta-analysis.

Stressors include disaster events like witnessing someone get hurt or die. Most of the papers included here describe these losses. More than 10,000 Nicaraguans were homeless, according to Caldera et al., and 2,000 people died during a hurricane there [27]. According to Huang et al., a flood in China resulted in 4,150 fatalities and more than 18 million displaced individuals [28]. Following a hurricane in Vietnam, the incidence rates of PTSD were found to range from 2.6% [34] to 90% [36] among students in the most seriously impacted city in Nicaragua. The variety of measuring tools, time points, included populations, types of disaster, and study features were potential causes for this range. Additionally, evaluation techniques developed for the western environment may not always be applicable to the cultures of low-income countries [34]. Poor health, high exposure, prior traumatic experiences, elderly age, home damage, seeing dead bodies, and seeing dead family members are investigated risk factors for PTSD [41]. Similar results from research on post-disaster mental health revealed a significant relationship between disaster and cognitive and mental health [43].

The older age of the study subjects may be the reason for the highest post-disaster anxiety rate which was found in one included study (84%) following the storm in Sri Lanka in 1978 [35]. Compared with this finding, Vietnam experienced the lowest post-disaster anxiety rate following a storm in 2006. Possible causes include the storm's less severe consequences compared to prior catastrophes, the low death toll, and the storm's successful evacuation [34]. One study showed the same findings which showed that exposure to both disaster-related traumatic events and to financial and social stressors influenced the duration of stress symptoms [44].

People were more likely to experience psychological distress after exposure to catastrophe occurrence if they had lower income levels, were economically inactive, were unemployed, or had pre-existing medical issues [21,22,24,25]. Similar results were observed in one study, which found that those with lower socioeconomic position experienced long-term psychological distress because of their encounter with flooding [45]. Moreover, this meta-analysis revealed that higher levels of anxiety immediately following disaster were linked to other monetary issues, such as a lack of insurance [25]. Lack of assistance from various authorities before, during, and after the disaster may be a factor in the psychological distress that has been experienced [46].

Rates of depression ranged from 5.9% following the typhoon in Vietnam in 2006 [34] to 81% following a hurricane in Nicaragua in 1998 [36]. The latter high rate could be brought on by a high prevalence of affected people, fatalities, and displacements. The potential causes of depressive illnesses were examined in two studies. They identified potential reasons as being poor health, prior traumatic events, female gender, damage to the home or belongings, fatalities, and unemployment [41,47].

The people exposed to disasters in the UK, India, Honduras, Sri Lanka, and Nicaragua had higher rates of common mental health issues, according to this meta-analysis. The effects of the catastrophes resulted in both short-term and long-term mental health issues. According to several studies, psychiatric disorders have become more prevalent because of the disaster [46,48]. The insufficient warning systems, water depth from floods, disruption of services, evacuation and relocation, a lack of post-disaster support, and social and economic inequities could all be contributing factors to the increased prevalence [49]. Contrarily, two studies in South Korea and China revealed a lower prevalence of mental disorder following floods [50,51]. The social vulnerability of women and their lack of experience with flooding were suggested as potential explanations for this discrepancy [46]. Furthermore, there is a connection between mental illness and the upheaval caused by catastrophes, which can lead to environmental degradation, a breakdown in social ties, and a loss of communal spirit. This lasted even a year after the event, indicating that displacement is a major secondary stressor that has a lasting impact on the outcomes of the mental health of those who experienced the tragedy [52,53].

Globally, the prevalence of mental illness has increased by roughly 17%. In the first year following a disaster, psychological morbidity frequently affects 30%-40% of the population, although a residual disease load is likely to continue to chromicize [54-56]. There is limited research on how disasters affect psychopathology [56].

The limitations of this study are that it only used self-reported health measures. Disaster studies are often carried out under exceedingly difficult situations. Typically, a vast region is affected, the exposure is dispersed unevenly, and some areas are possibly unreachable. The measuring instruments that have been created and validated in a western setting may not adequately reflect the burden of disease in less developed countries of the world [34]. The search only incorporated published works and a few selective sources. The major strength, however, is the comprehensive and effective search strategies. We had low risk publication bias. The study's overall moderate quality and inclusion of 48170 participants are two further strengths.

## Conclusions

Overall, most of the studies showed a connection between disaster and various ways in which mental health can deteriorate. This is concerning since it is expected that there will be an increase in mental illness soon because of extreme weather events. Natural catastrophe health consequences are not likely to be spread evenly or randomly among communities. In our meta-analysis, the “medium human development countries” were identified as having the greatest prevalence rate of mental health disorders. However, after the disastrous events, the “very high human development” and “high human development” countries had a greater prevalence rate of mental health illnesses as well. Nevertheless, the disparity between developed and developing nations needs to be addressed given that lower-income countries continue to be disproportionately affected by the devastating disasters brought on by climate change.

The status of the vulnerable population affected by the disaster can be addressed by increased community resilience, better access to healthcare services, and an adequate mitigation plan. Future research on other climate-related effects and broader mental health consequences may minimize the knowledge gaps.
